# Supplementary material for: Characterization and Genomic Analyses of dsDNA Vibriophage vB_VpaM_XM1, Representing a New Viral Family
Source: Mar Drugs. 2024 Sep 21;22(9):429. doi: 10.3390/md22090429 (PMC11432961; doi:10.3390/md22090429)
Supplement: Supplementary file 1 [file marinedrugs-22-00429-s001.zip › Supplementary Files.pdf]

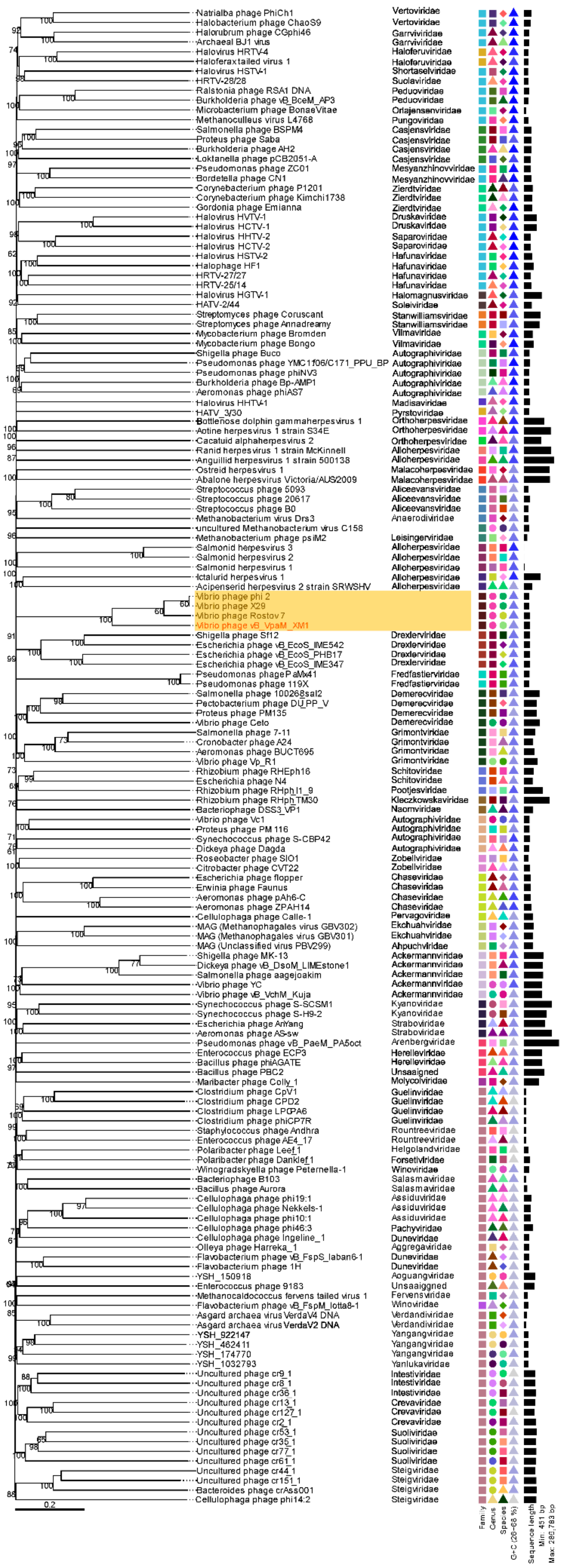

Figure S1: Genome BLAST distance phylogeny (GBDP) tree of 154 virus genomes. Based on

nucleotide sequences, the GBDP tree is reconstructed by VICTOR.

Figure S2: genome phylogenetic tree based on the nucleotide of the terminase large submit (A) and major capsid protein (B), showing the relationships between phage vB_VpaM_XM1 and other nearest phages.

Table S1: Strains used in the host-range test and their susceptibility to XM1.

Bacterial species	Strain	Isolation source	Susceptibility to phage vB_VpaM_XM1
Vibrio owensii DY05(T)	JL1211[a]	Bei bu wan	-
Vibrio azureus LC2-005(T)	JL1214[a]	Bei bu wan	-
Vibrio campbellii ATCC 25920(T)	JL2671[a]	Western pacific	-
Vibrio owensii DY05(T)	JL2663[a]	Western pacific	-
Vibrio alginolyticus ATCC 17749(T)	JL2674[a]	Western pacific	-
Vibrio owensii DY05(T)	JL2918[a]	South China Sea	-
Vibrio xuii LMG 21346(T)	JL2919[a]	South China Sea	+
Vibrio owensii DY05(T)	JL3187[a]	Xisha	-
Vibrio campbellii CAIM 519(T)	JL3506[a]	Guishan Island	-
Vibrio campbellii CAIM 519(T)	JL3507[a]	Guishan Island	-
Vibrio antiquarius Ex25(T)	JL3524[a]	Guishan Island	-
Vibrio neocaledonicus NC470(T)	JL3539[a]	Guishan Island	-
Vibrio neocaledonicus NC470(T)	JL3544[a]	Guishan Island	-
Vibrio neocaledonicus NC470(T)	JL3545[a]	Guishan Island	-
Vibrio neocaledonicus NC470(T)	JL3546[a]	Guishan Island	-
Vibrio mytili CAIM 528(T)	JL3548[a]	Guishan Island	-
Vibrio rotiferianus LMG 21460(T)	JL3557[a]	Guishan Island	-
Vibrio neocaledonicus NC470(T)	JL3558[a]	Guishan Island	-
Vibrio azureus NBRC 104587(T)	JL3560[a]	Guishan Island	-
Vibrio proteolyticus NBRC 13287(T)	JL3562[a]	Guishan Island	-
Vibrio neocaledonicus NC470(T)	JL3563[a]	Guishan Island	-
Vibrio fortis LMG 21557(T)	JL3565[a]	Guishan Island	-
Vibrio azureus NBRC 104587(T)	JL3577[a]	Guishan Island	-
Vibrio neocaledonicus NC470(T)	JL3703[a]	Guishan Island	-
Vibrio inhibens BFLP-10(T)	JL3707[a]	Guishan Island	-
Vibrio hyugaensis 090810a(T)	JL3710[a]	Guishan Island	-
Vibrio tubiashii ATCC 19109(T)	JL3833[a]	Xiamen Coastal area	-
Vibrio parahaemolyticus	MCCC 1H00029[b]	Food	+
Vibrio parahaemolyticus	LMG 16872[b]	Shrimp	-
Vibrio parahaemolyticus	MCCC 1H00058[b]	Seawater	-
Vibrio parahaemolyticus	MCCC 1H00801[b]	Kelp	-
	MCCC 1A11655[b]	Litopenaeus vannamei hepatopancreas	-

Vibrio parahaemolyticus	MCCC 1A16578[b]	Sediment/Deep dark sediment	-
Vibrio parahaemolyticus	MCCC 1A17892[b]	Sediment/Kandelia rhizosphere sediment	-
Vibrio parahaemolyticus	MCCC 1A16283[b]	Sediment/intertidal sediment	-
Vibrio parahaemolyticus	MCCC 1K02692[b]	Water sample/bottom sea water	-
Vibrio parahaemolyticus	Wa-1801[c]	Surface seawater,Yellow sea, China	-
Idiomarina sp	Wa-1802[c]	Surface seawater,Yellow sea, China	-
Pseudoalteromonas sp	Wa-1803[c]	Surface seawater,Yellow sea, China	-
Pseudoalteromonas marina	AbY-1805[c]	Cells of Haliotis discus hannai Ino, China	-
Vibrio natriegens	AbY-1901[c]	Cells of Haliotis discus hannai Ino, China	-
Vibrio owensii	AbY-1902[c]	Cells of Haliotis discus hannai Ino, China	-
Vibrio mediterranei	AbY-1903[c]	Cells of Haliotis discus hannai Ino, China	-
Photobacterium jeanii	AbG-1906[c]	Cells of Haliotis discus hannai Ino, China	-
Vibrio chagasii	AbG-1907[c]	Cells of Haliotis discus hannai Ino, China	-
Vibrio chagasii	AbG-1908[c]	Cells of Haliotis discus hannai Ino, China	-
Shewanella waksmanii	ScY-2105[c]	Cells of Mizuhopecten yessoensis, China	-
Vibrio alginolyticus	ScY-2106[c]	Cells of Azumapecten farreri, China	-
Vibrio alginolyticus	ScY-2107[c]	Cells of Azumapecten farreri, China	-
Photobacterium rosenbergii	ScY-2109[c]	Cells of Azumapecten farreri, China	-
Vibrio atlanticus	ScG-2111[c]	Cells of Azumapecten farreri, China	-
Vibrio celticus	WaY-2217[c]	Coastal seawater, China	-
Vibrio parahaemolyticus	AbY-2219[c]	Cells of Haliotis discushannai Ino, China	-
Vibrio crassostreae	WaY-2220[c]	Larval rearing water,China	-
Vibrio alginolyticus	WaG-2229[c]	Larval rearing water,China	-
Vibrio parahaemolyticus	MCCC1A14388[c]	Shrimp farm water, China	-
Vibrio natriegens	MCCC1K03861[c]	Sediment of oyster farming area, China	-
Vibrio natriegens	NBRC15636[c]		-
Vibrio natriegens			

+， susceptible; -, resistant

a: Strains were obtained from Collection Center of Marine Bacteria (CCMB, China)

b: Strains were purchased from the Marine Culture Collection of China (MCCC, China)

c: Strains used in previous study (Li X, Liang Y, Wang Z, et al. Isolation and Characterization of a Novel Vibrio natriegens—Infecting Phage and Its Potential Therapeutic Application in Abalone Aquaculture[J]. Biology, 2022, 11(11): 1670.)
